# Beyond the numbers: United European Gastroenterology journal's mission to build a community and connect

**DOI:** 10.1002/ueg2.12447

**Published:** 2023-07-29

**Authors:** Iris J. M. Levink, Alberto Balduzzi, Joost P. H. Drenth

**Affiliations:** ^1^ Department of Gastroenterology and Hepatology Erasmus University Medical Centre Rotterdam The Netherlands; ^2^ Department of Surgery, Dentistry, Paediatrics and Gynaecology Unit of General and Pancreatic Surgery The Pancreas Institute Verona University of Verona Verona Italy; ^3^ Department of Gastroenterology and Hepatology Radboud University Nijmegen Medical Centre Nijmegen The Netherlands

**Keywords:** basic, clinical, digestive diseases, European, gastroenterology, hepatology, journal, open access, research, translational

## INTRODUCTION

The United European Gastroenterology (UEG) is a community of over 50,000 professionals dedicated to advancing the prevention and care of digestive diseases in Europe by providing education and supporting research. The journal, launched in 2013, is a cornerstone of UEG. At the time of its inception, the journal has formulated its mission as an international forum for research in gastroenterology, publishing original articles that investigate basic, translational, and clinical studies of interest to gastroenterologists and researchers in related fields. It aimed to position itself in the top 15% of gastroenterology and hepatology journals, providing authoritative, high‐quality, open access (since 2021) platform. In this editorial, we will provide a critical appraisal of the journal's success and challenges to date whilst trying to achieve these goals. We hope to offer a comprehensive picture of the UEG journal's impact and influence within the field of gastroenterology.

## AUTHOR EXPERIENCE

The success of a journal partly comes down to the willingness of the authors to publish their high‐quality research. This willingness often depends on the impact factor, as a measure of citation rate, but also the commitment to the journal, the experience of the authors during the submission process (website handling, costs, duration), and the expected visibility of the article. The UEG journal aims to attract top talent and cutting‐edge research in the field of gastroenterology by providing a supportive and high‐quality publishing experience for authors, whilst maintaining rigor and quality and providing timely and constructive feedback to authors. The countries that submitted the most articles in 2022 were China, Germany, Italy, the Netherlands, and the United Kingdom. We foster a dynamic research community, hoping to increase our diversity and global reach.

**FIGURE 1:**
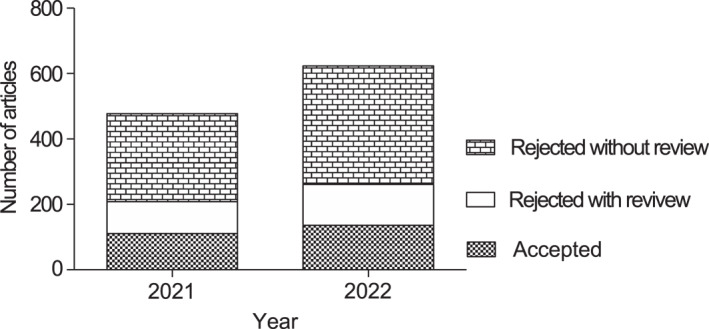
The absolute number of accepted and rejected articles listed in the year the final decision was taken. The acceptance rate was 21.8% in 2022, a decrease from 23.3% in 2021.

In 2022, the UEG journal saw a significant increase in the number of submissions, with a 32.2% rise compared to the previous year (Figure 1). This stands in contrast to the overall trend across all Wiley journals in the Gastroenterology & Hepatology subject area, which saw a slight decrease (−3.1%) in submissions during the same period. The acceptance rate for submissions in 2022 was 21.8%, which was slightly lower as compared to 2021 (23.3%; Figure 1). This suggests that the journal is maintaining a high bar for quality, even as it attracts an increasing volume of submissions.

The journal also made progress in reducing the time from submission to acceptance, with a median of 43 days in 2022, down from 54 days in 2021, excluding editorials or invitations that were generally accepted before peer review. Striking was the response rate of reviewers. The journal sent out 1343 review invitations in 2022, with 696 accepted and 618 completed reviews within a median of 12 days. This high response rate may be due to the participation of young researchers on the editorial board that shows a strong willingness to review, as well as the overall involvement in UEG of other researchers and the possibility of claiming their review of Publons.

After the acceptance of the paper, publications follow quickly. In 2022, the average number of days from receipt at Wiley to Early View for United European Gastroenterology Journal was 19 (median 14), as compared to the Medical Sciences average of 27 (median 18) and the Wiley average of 26 (median 18). This demonstrates the journal's commitment to publishing research in a timely manner, which is an important factor for both authors and readers.

Moreover, Wiley recently launched a Wiley AI Editing Pilot program, which may be a welcome addition for authors with English as their second language, as it provides additional support during the submission and reviewing process. In short, this program will edit manuscripts with an intelligent, AI‐driven tool to ensure that the manuscript is free of any major grammatical or syntactical errors before submission to UEG Journal.

## READERSHIP

**FIGURE 2:**
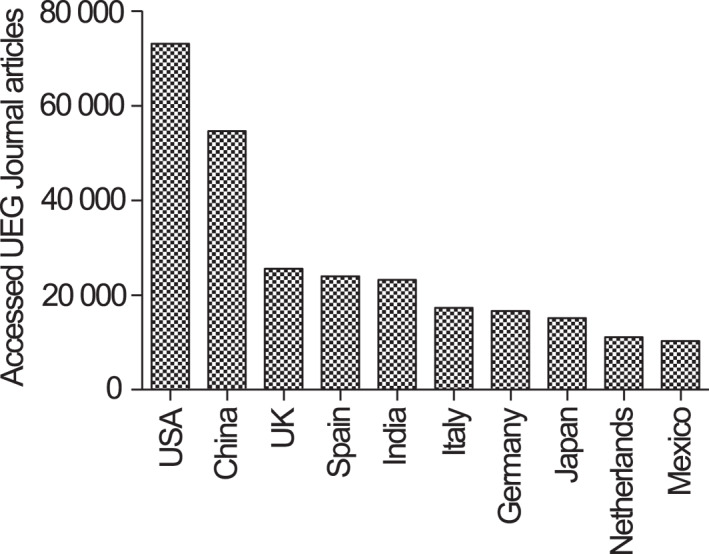
This chart shows the top 10 countries/regions from which articles in the United European Gastroenterology (UEG) journal were accessed via the Wiley Online Library in 2022.

One objective measure of a journal's impact is the number of views, as this indicates the extent to which its research is being accessed and utilized by the wider community. Based on data from 2022, eight out of ten most viewed articles in the UEG journal were guidelines with 5000–15,000 views each.^1^, ^2^, ^3^, ^4^, ^5^, ^6^, ^7^, ^8^, ^9^, ^10^ This suggests that there is a high demand for authoritative guidance in the field of gastroenterology and that the UEG Journal is fulfilling an important role in disseminating this crucial information to its readers. On average, articles published in the UEG journal in 2022 received 931 views. While this may seem modest compared to some other well‐established journals, it is important to note that the UEG journal is a specialized journal that caters to a particular audience (the Wiley average in the same subject area is 428 views). In 2022, the number of UEG Journal Online Library views has risen by 17.6% compared to 2021. The journal is gaining traction amongst its target audience and is becoming increasingly recognized as a valuable source of information in the field of gastroenterology. The UEG journal's impact is truly global, as most of its traffic, as measured in Online Library views, came from five main countries: United States, China, the United Kingdom, Spain, and India (Figure 2).

## SOCIAL MEDIA ENGAGEMENT

The increase in the number of views may be attributable to its strong emphasis on social media. UEG Journal Twitter (@UEGJournal) account managed to generate significant engagement and has now reached 9000 followers (as per March 2023). UEG journal's impact is not limited to its immediate readership but extends through its online presence as well. The journal is hosted on the Wiley Online Library platform, which provides global open access to its articles and research not limited to traditional academic circles. By providing accessible, high‐quality research that is relevant to a broad range of stakeholders, the UEG journal can continue to expand its impact within the field of gastroenterology and beyond.

Twitter mentions have been the second most impactful altimetric for the UEG journal (11%), with Google Search driving the most traffic (17.4%). Each article published in the UEG Journal has undergone extensive search engine optimization (SEO) by the editorial team aiming to make its content more discoverable online. This percentage may implicate that this SEO has been effective.

## UEG JOURNAL METRICS

The UEG journal continues to show strong performance in terms of citation impact, with a CiteScore (Scopus) of 7.9 and a Journal Citation Indicator (Clarivate) of 0.9. Additionally, the Journal Impact Factor (Clarivate) for 2021 was 6.866 (5‐year IF 5.424).

## CONCLUSION

The UEG Journal is on a mission to provide an authoritative, high‐quality, international, and trusted platform for all research in gastroenterology. Overall, metrics demonstrate that the journal continues to grow in popularity and impact, even in a highly competitive field, having reached its goal of positioning itself in the top 15% of gastroenterology and hepatology journals. The UEG journal is successfully reaching and engaging its target audience through a combination of effective SEO and increasing social media outreach. With future investments in online and digital media, we aim to reach an even wider audience.

The role of guidelines within the UEG Journal is increasing. Based on the current metrics, these are viewed and appreciated. The UEG quality of care committee has played an important role in this by stimulating the generation of high‐quality guidelines and publication in the UEG Journal. The UEG editorial board, in collaboration with the quality‐of‐care committee, aims to further increase the quality of guidelines by formulating criteria and extensive peer review by reviewers who have undergone training in the guideline formulation. This renewed focus on the quality of guidelines may further cement the UEG Journal reputation as a valuable resource for gastroenterologists and other medical professionals in the field of digestive diseases.

Looking ahead, the UEG Journal is well‐positioned for continued growth and success. The journal's upward trajectory^11^ is a testament to the dedication and hard work of its editorial team, reviewers, authors, and readers. With a steadfast commitment to excellence and a focus on innovation, the UEG Journal will undoubtedly continue to lead the way in advancing the prevention and care of digestive diseases in Europe and beyond.

## CONFLICT OF INTEREST STATEMENT

All authors are members of the United European Gastroenterology Journal's editorial board.

## Data Availability

Data sharing is not applicable to this article as no new data were created or analyzed in this study.
